# Interaction of Remalan Brilliant Blue R dye with *n*-alkyltrimethylammonium chloride surfactants: conductometric and spectroscopic investigations

**DOI:** 10.1098/rsos.241344

**Published:** 2025-02-19

**Authors:** Ashraf El-Hashani, Manish Kumar Sah, Khaled Edbey, Rehab Gaber Ismael, Dileep Kumar, Ajaya Bhattarai

**Affiliations:** ^1^ Department of Chemistry, Faculty of Science, University of Benghazi, Benghazi 16063, Libya; ^2^ Department of Chemistry, Mahendra Morang Adarsh Multiple Campus, Tribhuvan University, Biratnagar 56613, Nepal; ^3^ Libyan Authority for Scientific Research, PO Box 80045, Tripoli, Libya; ^4^ Laboratory for Chemical Computation and Modeling, Institute for Computational Science and Artificial Intelligence, Van Lang University, Ho Chi Minh City, Vietnam; ^5^ Faculty of Applied Technology, School of Technology, Van Lang University, Ho Chi Minh City, Vietnam; ^6^ Department of Chemistry, Indian Institute of Technology Madras, Chennai 600036, India

**Keywords:** *n*-alkyltrimethylammonium chloride, Remalan Brilliant Blue R, UV spectroscopy, conductivity, Gibbs free energy

## Abstract

Micellization behaviour, thermodynamics and dye–surfactant interactions are the main topics of this investigation into the relationship between Remalan Brilliant Blue R (RBBR) dye and *n*-alkyltrimethylammonium chloride (C_*n*_TAC; *n* = 12, 14, 16 and 18) surfactants. Conductometric analysis revealed that the critical micelle concentration decreased with increasing alkyl chain length, suggesting that longer chains formed better micelles. Due to electrostatic interactions, spectrophotometric analyses revealed notable alterations in RBBR absorption following contact with C_*n*_TAC. Benesi–Hildebrand and Scott equations were used to compute the binding constant (*K*_b_), which increased with chain length, indicating stronger dye–micelle interactions. The spontaneous interactions between RBBR and micelles were confirmed by thermodynamic analysis, which showed negative Gibbs free energy values (Δ*G*). The stability of dye–micelle complexes is attributed to hydrophobic forces, as seen by the greater negative Δ*G* that was produced by longer alkyl chains. Interestingly, increased surfactant concentrations changed equilibrium and decreased dye adsorption by breaking up pre-formed RBBR–C_*n*_TAC complexes. These results highlight how important the length of the alkyl chain is for micelle formation, thermodynamic parameters and dye–surfactant interactions. As the length of the chain grew, the sequence of RBBR binding strength was found to be C_12_TAC < C_14_TAC < C_16_TAC < C_18_TAC.

## 1. Introduction

Among the approximately 100 000 synthetic dye structures, more than 3600 are currently commercially marketed. Anion is the active moiety in azo dyes, which is notable as they comprise almost half of all synthetic dyes used in commercial applications [1].

The cationic surfactant *n*-alkyltrimethylammonium chloride (C_*n*_TAC) is classified by its positively charged head groups. These surfactants have a significant affinity for negatively charged surfaces, which makes them easier to adsorb onto materials such as cell membranes, fabrics and hair. Surfactants perform various tasks including nourishing hair, drug delivery, chemical kinetics, softening cloth, etc., and have antimicrobial qualities [2–8]. C_*n*_TAX (X = Cl^−^ or Br^−^) are used mainly as detergents, but they are also widely used with many additive systems in many industrial areas and research intentions using several techniques [9–21].

**Figure 1.**
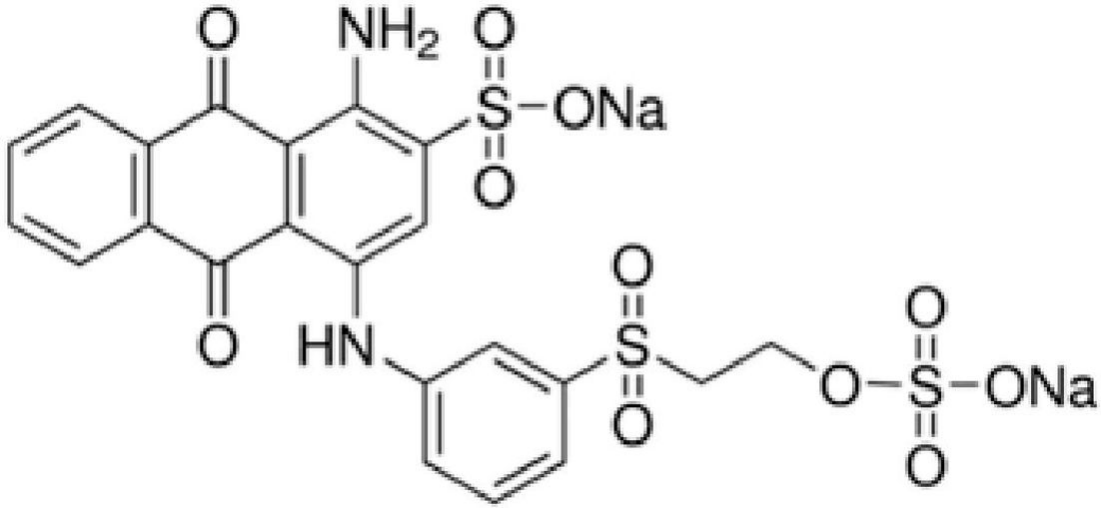
Molecular structure of RBBR.

Remalan Brilliant Blue R (RBBR; figure 1) is a synthetic dye that is used widely for a variety of purposes, particularly in the textile industry to colour garments. It belongs to the azo dye class and is known for its vivid and strong hues. When dyeing textiles to produce vibrant and long-lasting blue hues, RBBR is a good choice because of its great colour fastness and usually bright blue powder appearance. In addition, RBBR is utilized in other sectors of the economy, including food, cosmetics and medicines. It is a colourant in products like beverages, cosmetics and medication compositions. RBBR is widely utilized to add colour to a variety of materials in various industries due to its stability and versatility [22,23].

The study of the interaction between RBBR and cetyltrimethylammonium chloride (CTAC) with respect to UV investigations and conductivity in relation to critical micelle concentration (CMC) is one topic that requires attention. Although most people are aware of the characteristics of CTAC and RBBR, little is known about how they interact specifically with UV light and how conductivity dynamics change in relation to CMC [24]. Examining how RBBR and CTAC react could contribute to the development of innovative materials for environmental monitoring, where the identification and elimination of pollutants are essential. The results may also have consequences for the pharmaceutical and biotechnology industries, as drug delivery systems and biological applications depend on a knowledge of the behaviour of dye–surfactant complex applying a variety of instrumental devices [25–38].

Buwalda *et al*. explored the interactions between a dye (methyl orange) and surfactants [39]. Their findings provide valuable information for our current investigation of RBBR.

The research focuses on conductivity dynamics and UV-induced interactions close to the CMC in order to fill the knowledge gap regarding the interactions between RBBR dye and CTAC. The precise behaviour of RBBR and CTAC, especially with regard to micelle generation and stability, is still poorly understood, despite the fact that dye–surfactant systems have been studied previously. The development of cutting-edge materials for environmental monitoring, including systems for identifying and removing pollutants, as well as applications in biotechnology, cosmetics and pharmaceuticals, are all impacted by this crucial gap. Using UV spectroscopy and conductivity tests to discover structural and electronic insights, the inquiry seeks to estimate binding constants, CMC values and thermodynamic parameters (∆*G*) in order to get a greater understanding of RBBR–C_*n*_TAC interactions across a range of alkyl chain lengths.

By filling this study gap, we can get a good understanding of how C_*n*_TAC and RBBR behave as a group, leading to new research avenues and optimization strategies. With regard to the interaction between C_*n*_TAC and RBBR, UV spectroscopy is a crucial area of research [40,41]. With the goal of improving colour and stability, RBBR–C_*n*_TAC complexes are useful in fields like cosmetic formulations and water cleanup. New research shows that surfactants can influence the way dye solutions absorb specific wavelengths of light, which can be observed through changes in their absorption spectra. Although some previous studies have investigated the interaction mechanism between different C_*n*_TAC with varying alkyl chain lengths (*n* = 12, 14, 16 and 18), more research on the RBBR–C_*n*_TAC system is still necessary [42].

This study aims to investigate the intriguing associations of RBBR and C_*n*_TAC variants that have different chain lengths (*n* = 12, 14, 16 and 18). The determination of the binding constants of these surfactant–dye complexes and CMC values provides insight into the self-assembly behaviour of these compounds, as well as the type and intensity of their interactions. The calculation of ∆*G* provides thermodynamic insight into molecular interactions. UV spectroscopic examination reveals structural modifications and electronic transitions that occur during complex formation. By combining spectral and conductometric studies, this integrated strategy seeks to bridge the existing understanding gap in C_*n*_TAC–RBBR interactions that could be used in a variety of sectors.

## 2. Experimental

### 2.1. Materials

In this study, four different surfactants were used: dodecyltrimethylammonium chloride (C_12_TAC) (shortest chain), tetradecyltrimethylammonium chloride (C_14_TAC), hexadecyltrimethylammonium chloride (C_16_TAC) and octadecyltrimethylammonium chloride (C_18_TAC) (longest chain). All were sourced from Merck, UK, with a purity of 99%. We also obtained anionic RRBR dye (Sigma-Aldrich) with a purity of 50%. For the preparation of the solutions, double-distilled water with a conductivity of less than 0.1 µS was utilized.

### 2.2. Methods

To generate surfactant solutions with varying concentrations, we diluted the corresponding stock solutions using double-distilled water. A stock solution of RBBR was prepared at a concentration of 1.0 × 10^–3^ M and subsequently diluted to a concentration of 1.0 × 10^–5^ M in order to achieve an absorbance range suitable for analysis. To measure conductivity, a conductivity meter (Mettler 226, Switzerland) was used. This meter had a probe made of stainless steel (cell) with a specific value (cell constant of 0.726 cm^−1^) that helps determine conductivity accurately. The meter was calibrated using standard solutions of sodium chloride, as described elsewhere [43]. By identifying the intersection point of two linear segments in the conductivity versus C_*n*_TAC concentration plot, the CMC values were determined [44–50]. The repeatability of the conductance measurements was found to be 0.5%. The samples were placed in a 10 mm wide silica cuvette for the measurements. To analyse the light absorbed by the samples, a UV–visible spectrophotometer (Biochrom Libra S50, UK) was used that works with a single light beam. The measurements were performed at room temperature, which was around 298.15 K.

## 3. Results and discussion

### 3.1. The use of electrical conductivity to determine critical micelle concentration values

The physical characteristics of the surfactant solution are plotted against surfactant concentration in the usual method of calculating the CMC, and the point of inflection on the plot is identified [51,52]. The conductometric technique detects changes in ion concentration in the solution and generates an inflection point in the conductivity data as a function of surfactant concentration [53–58]. The specific conductivity (*ĸ*) measurements of C_*n*_TAC at 298.15 K for *n* = 12, 14, 16 and 18 are shown in electronic supplementary material, figures S1–S4. Micelle formation initiates at concentrations surpassing CMC, and this concentration can be pinpointed by a sudden change in the rate of increase (change in slope) or a distinct bend (inflection point) in the plot generated by the electrical conductivity measurements. The presence of higher concentrations of C_*n*_TA^+^ (cationic surfactant) and Cl^−^ (counterions) in the solution leads to increased conductivity below the CMC. Above the CMC, the presence of a higher number of surfactant molecules in the solution promotes the formation of micelles. However, the concentration of monomers in the solution remains relatively unchanged. However, micelles, compared to monomers, exhibit slower diffusion and larger size, resulting in reduced efficiency as charge carriers. Consequently, as the surfactant concentration increases, the CMC and conductivity increase more gradually.

**Figure 2.**
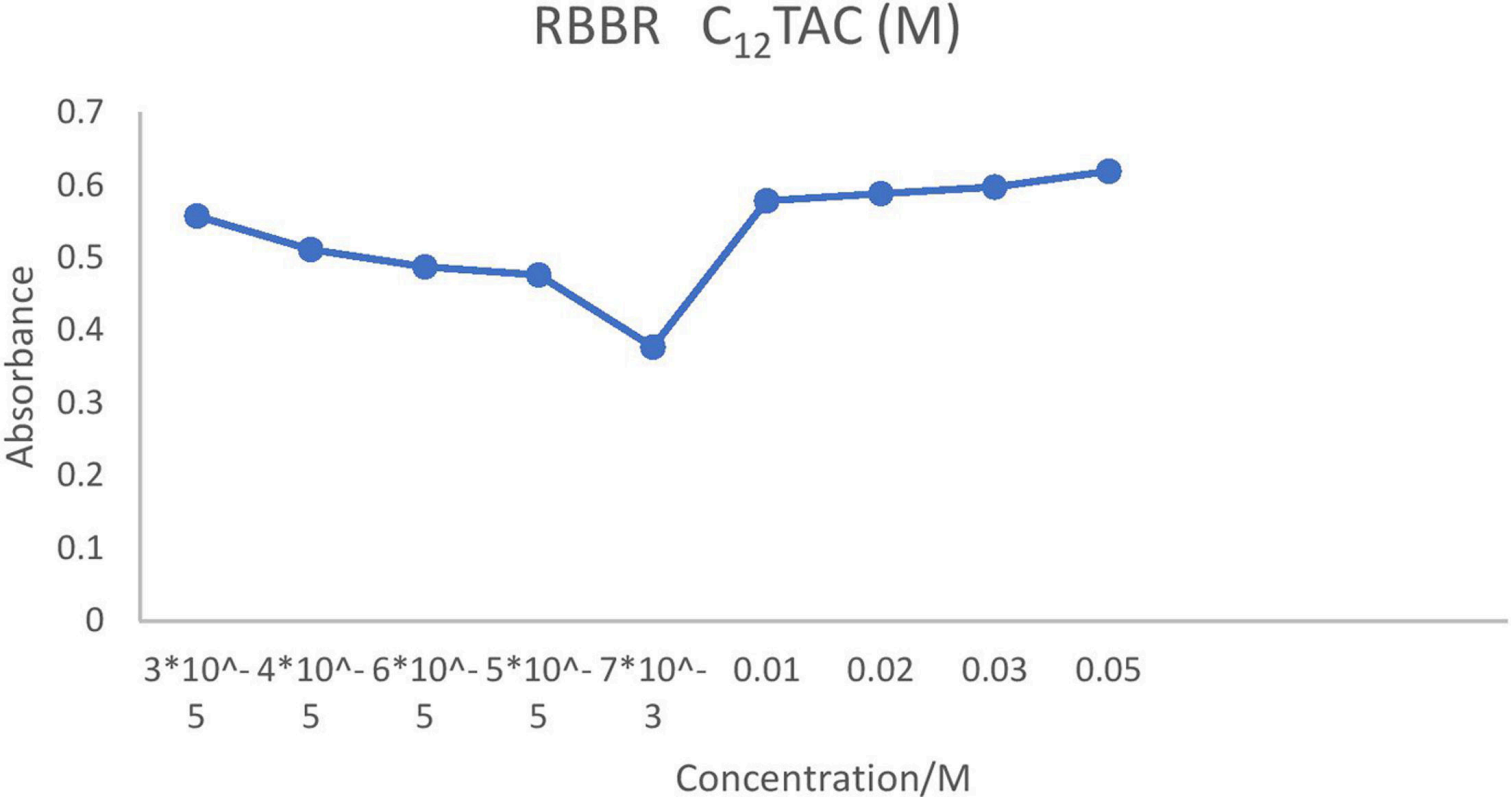
CMC of RBBR (3 × 10^−5^ M) at different concentrations of C_12_TAC.

**Figure 3.**
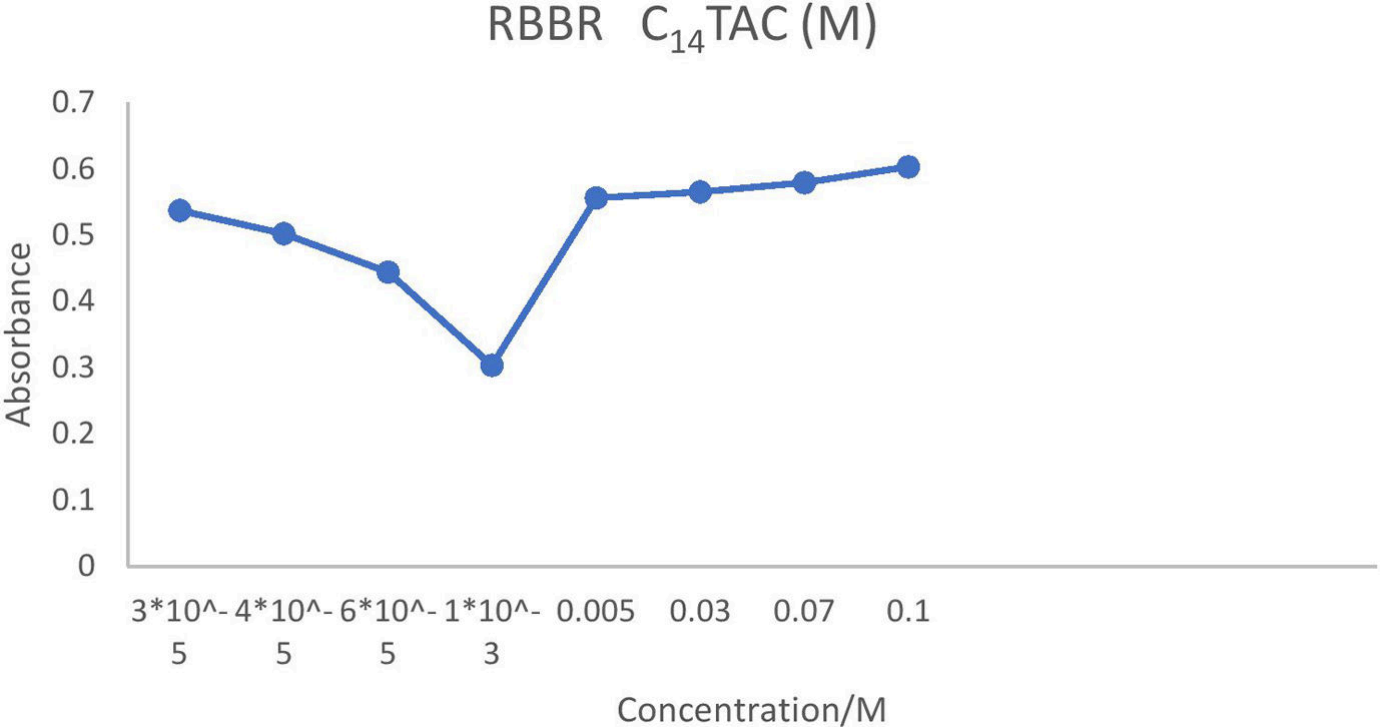
CMC of RBBR (3 × 10^−5^ M) at different concentrations of C_14_TAC.

**Figure 4.**
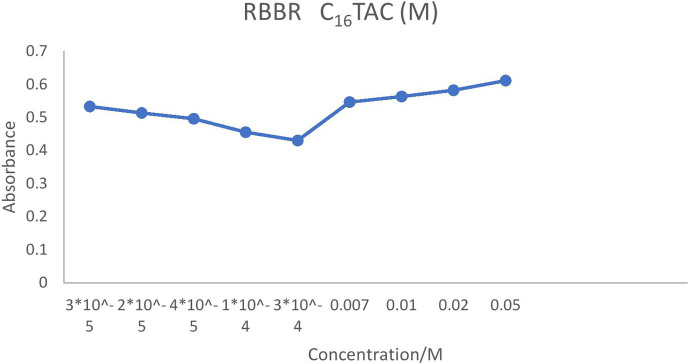
CMC of RBBR (3 × 10^−5^ M) at different concentrations of C_16_TAC.

**Figure 5.**
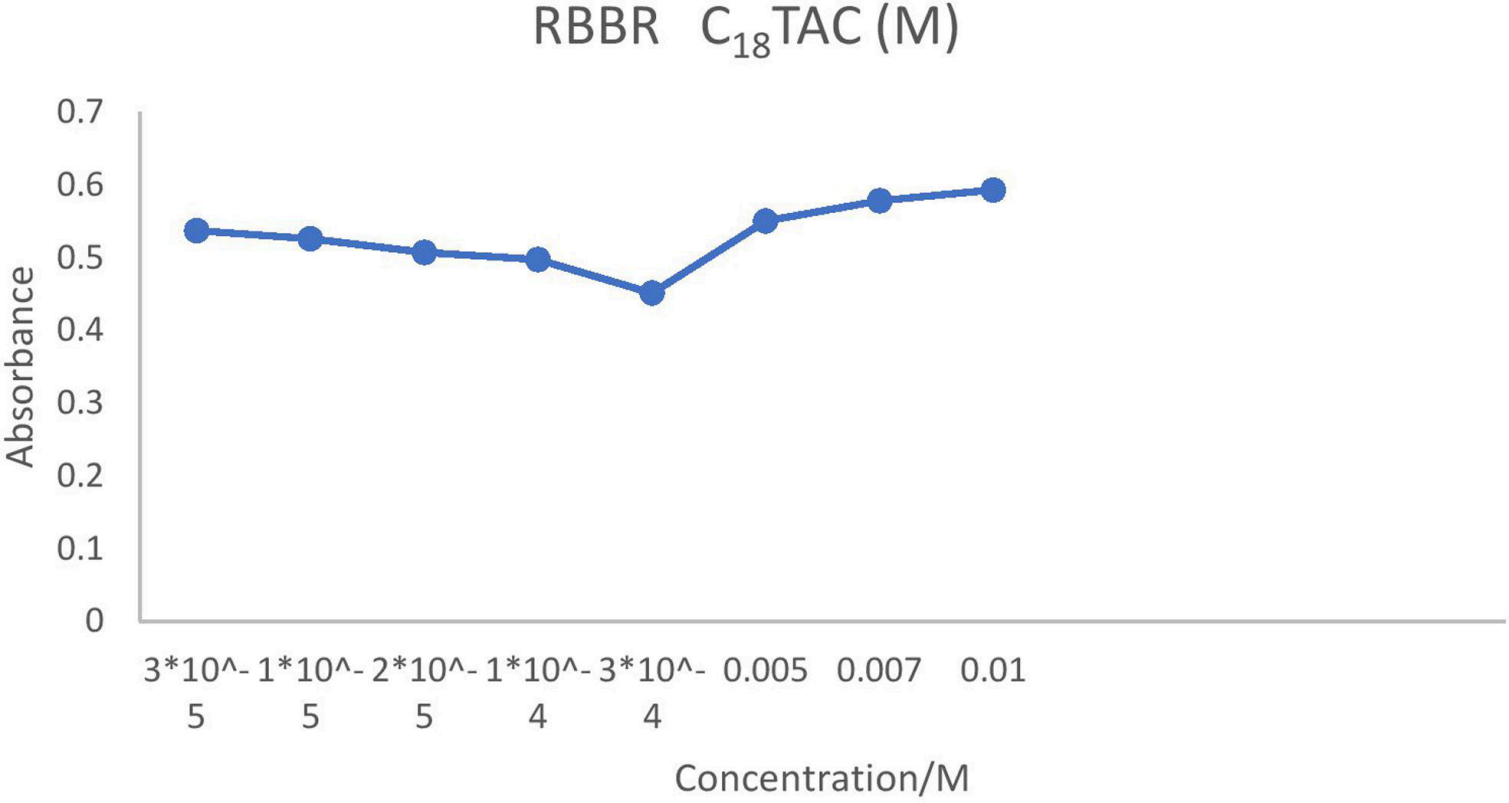
CMC of RBBR (3 × 10^−5^ M) at different concentrations of C_18_TAC.

On the contrary, ionic charges are lost above the CMC as counterions are restricted to the micellar surface. For instance, the conductivity increased more slowly when the concentration of C_12_TAC dropped from 5 × 10^–3^ to 5 × 10^–4^ at 6 × 10^–5^ and 3 × 10^–5^ M. The slower rise in solution conductivity is caused by the need for ionic monomers in micelle formation as well as the development of a second layer of ions around the micelle. Above the CMC, conductivity gradually increases as surplus cations preferentially form micelles rather than remain as free monomers. Lower concentrations cause the hydrophobic tails of C_*n*_TAC to come into contact with water molecules, which increases their surface energy and prevents tightly packed molecules which are presented in figures 2–5.

Thus, it is likely that this mechanism is responsible for the decrease in the CMC value of C_*n*_TAC as *n* (carbon chain length) increases.

Increasing the concentration of C_*n*_TAC causes the hydrophobic tails to pack closer together, resulting in a decrease in the free energy of the system and minimizing the amount of water to which surfactant molecules are exposed. Due to the increasing hydrophobicity of this phenomenon, micelle production is easier, and the CMC value decreases [59]. Indeed, it is important to consider the influence of the surrounding environment when it comes to micelle formation. For example, the type of ions present (like bromide) and the overall electrical charge of the solution (ionic strength) can influence the CMC of C_*n*_TAC. Higher ionic strength can help micelles form by reducing the electrical repulsion between the positively charged ends of the C_*n*_TAC molecules. This screening effect allows for closer proximity and more favourable interactions among surfactant molecules, thereby promoting micelle formation [60].

Micelle production is more likely to occur at higher concentrations of C_*n*_TAC because of increased hydrophobic interactions between surfactant molecules, resulting in a reduction in ∆*G*. This increased thermodynamic driving force indicates that the micellar core’s ability to solubilize hydrophobic materials has improved. These results have potential applications in the fields of chemistry, materials science and biotechnology making use of efficient and reasonably priced surfactants such as C_*n*_TAC.

A higher concentration of C_*n*_TAC improves hydrophobic interactions, which lowers free energy and minimizes exposure of the surfactant to water. Micelle production is made easier and the CMC is decreased as a result. Environmental factors like ion type and strength have a big influence on this process. Greater ionic strength encourages stable micelle formation by lowering repulsion between positively charged C_*n*_TAC heads. Stronger thermodynamic driving forces, which improve micelle solubilization of hydrophobic materials, are shown by the lower ∆*G*. These results demonstrate the promise of C_*n*_TAC’ for low-cost materials science and biotechnology applications.

In addition to the CMC, another indicator of micelle formation is the degree of micelle ionization (*α*) as equation (3.1):


(3.1) $$\alpha =S_{1}/S_{2},$$

where *α* reflects some property related to the interaction between the C_*n*_TAC surfactant and the solution. *S*_1_ represents the slope of the specific conductivity before the CMC is reached. *S*_2_ represents the slope of the specific conductivity after the CMC is reached.

These slopes provide information on the change in conductivity with respect to the concentration of C_*n*_TAC and can be used to analyse the behaviour and properties of the surfactant system. In order to describe the ∆*G* associated with micelle generation, equation (3.2) can be used [61,62]:


(3.2) $$\Delta G=(2-\alpha )RT\ln X_{cmc},$$

where *T* represents temperature (K), *R* represents the universal gas constant (8.314 J mol^−1^ K^−1^) and *X*_cmc_ represents the surfactant mole fraction at the CMC.

Electronic supplementary material, table S1, provides the CMC and *α* of C_*n*_TAC for various concentrations (1.4 × 10^–2^, 3.7 × 10^–3^, 9 × 10^–4^ and 6.1 × 10^–4^ M) at 298.15 K. This table provides valuable information on the behaviour and properties of C_*n*_TAC surfactants under different concentration conditions. The spontaneous formation of micelles is driven by a key force: hydrophobic interactions. These interactions occur between the tails of the surfactant molecules, which dislike water (hydrophobic). These interactions result in the aggregation of the hydrophobic tails, effectively minimizing their contact with the surrounding aqueous environment. By reducing the exposure of hydrophobic constituents to water molecules, the method aims to decrease the unfavourable interactions between hydrophobic regions and water.

This enables the surfactant molecules to self-assemble into micelles, where they arrange themselves with hydrophobic tails forming a core and hydrophilic groups forming a shell. This structure allows the coexistence of hydrophobic and hydrophilic components within micelles, promoting their stability [63].

A higher concentration of C_*n*_TAC improves hydrophobic interactions, which lowers free energy and minimizes exposure of the surfactant to water. Micelle production is made easier and the CMC is decreased as a result. Environmental factors like ion type and strength have a big influence on this process. Greater ionic strength encourages stable micelle formation by lowering repulsion between positively charged C_*n*_TAC heads. Stronger thermodynamic driving forces, which improve micelle solubilization of hydrophobic materials, are shown by the lower ∆*G*. These results demonstrate the promise of C_*n*_TAC for low-cost materials science and biotechnology applications.

The values of Δ*G* for pure C_*n*_TAC that we obtained in our investigation are closely correlated with those reported in the literature [64–66]. Although *α* values are not known, our results show that *α* and Δ*G* consistently decrease as the length of the alkyl chain (*n*) increases from 12 to 18. This observation highlights the important role of the alkyl group in micelle production.

### 3.2. Absorption study of C_*n*_TAC and RBBR

**Figure 6.**
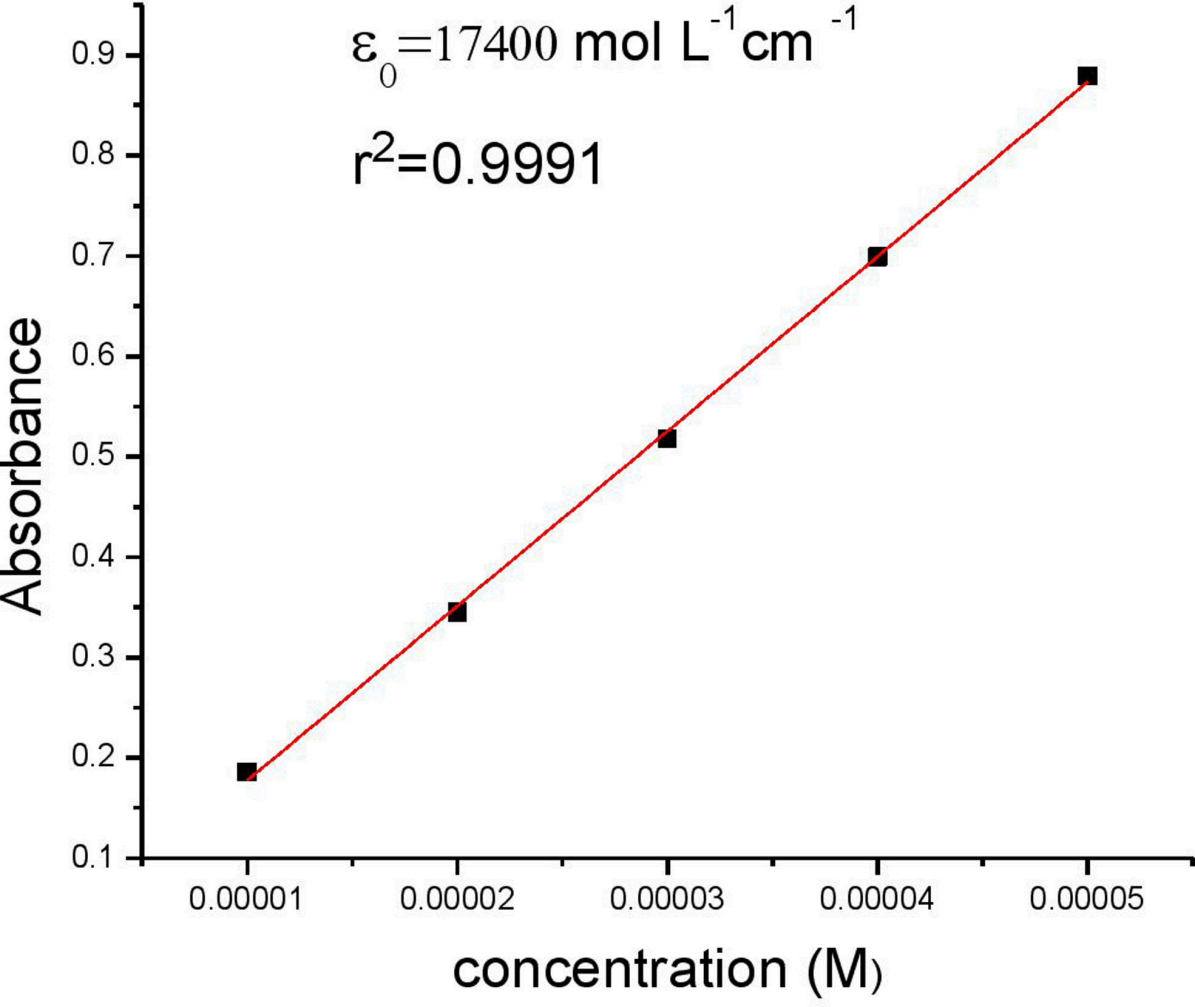
Obtaining the molar extinction coefficient (*ε*_0_) of RBBR.

The absorption spectrum of RBBR in aqueous solution with a concentration of 1.0 × 10^–5^ M exhibits a strong absorption peak at 597 nm. The result is supported by figure 6 showing a good linear connection (*R*^2^ = 0.9998) and the dye’s molar extinction coefficient (*ε*_0_) at this wavelength is determined to be 17 400 mol l^−1^ cm^−1^.

**Figure 7.**
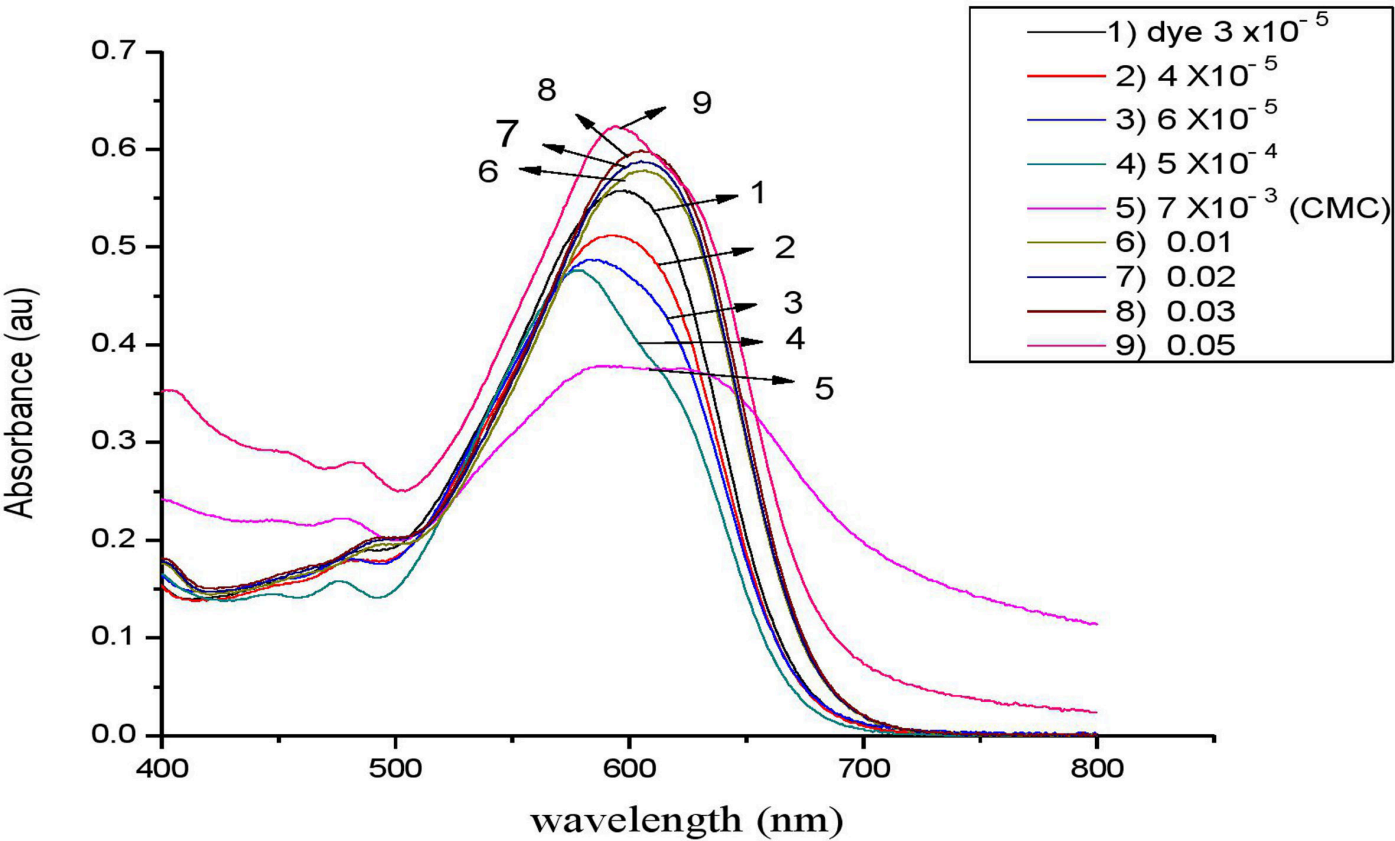
UV–visible absorption spectra of RBBR (7 × 10^−3^ M) associated with C_12_TAC at different concentrations.

**Figure 8.**
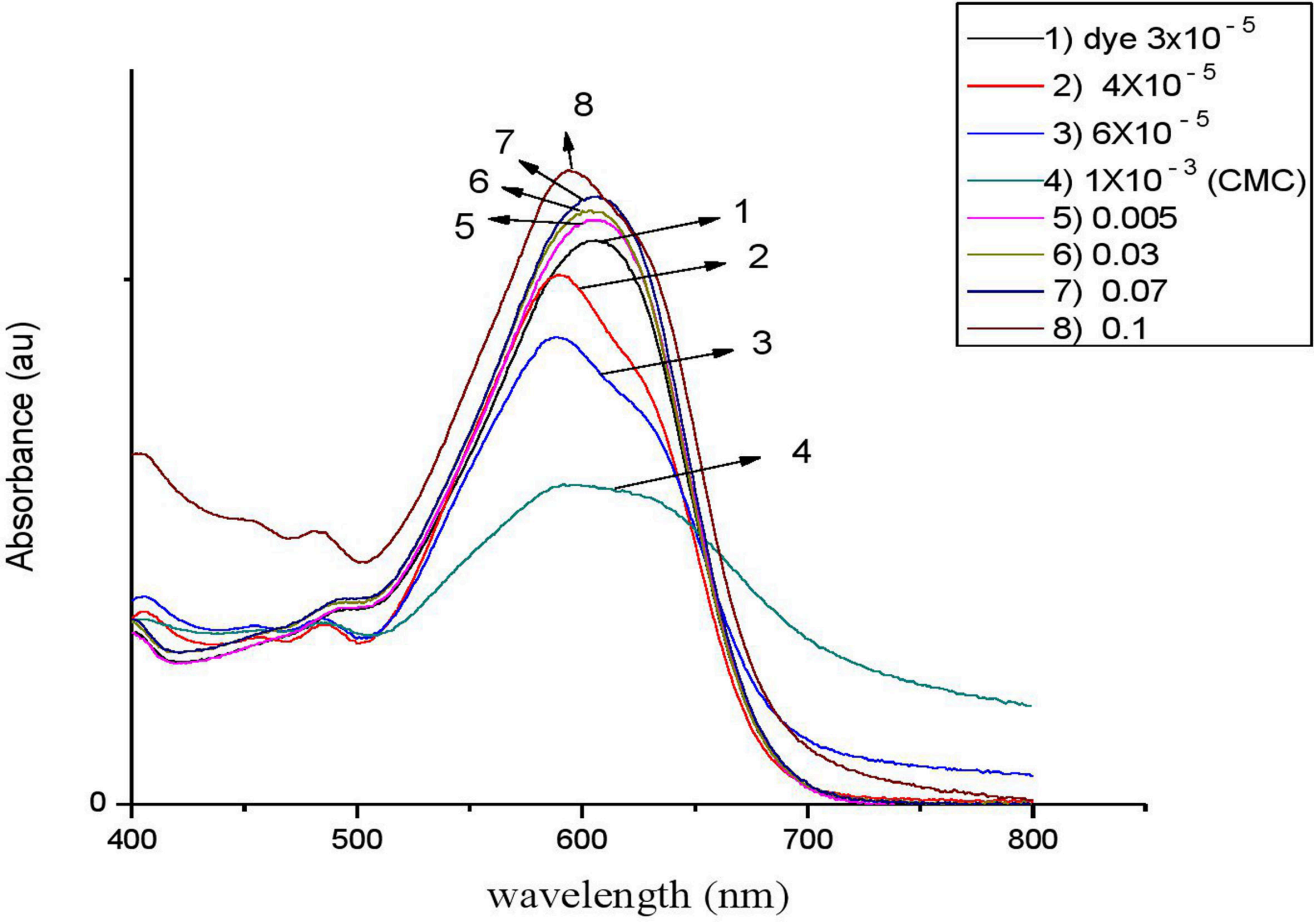
UV–visible absorption spectra of RBBR (1 × 10^−3^ M) associated with C_14_TAC at different concentrations.

**Figure 9.**
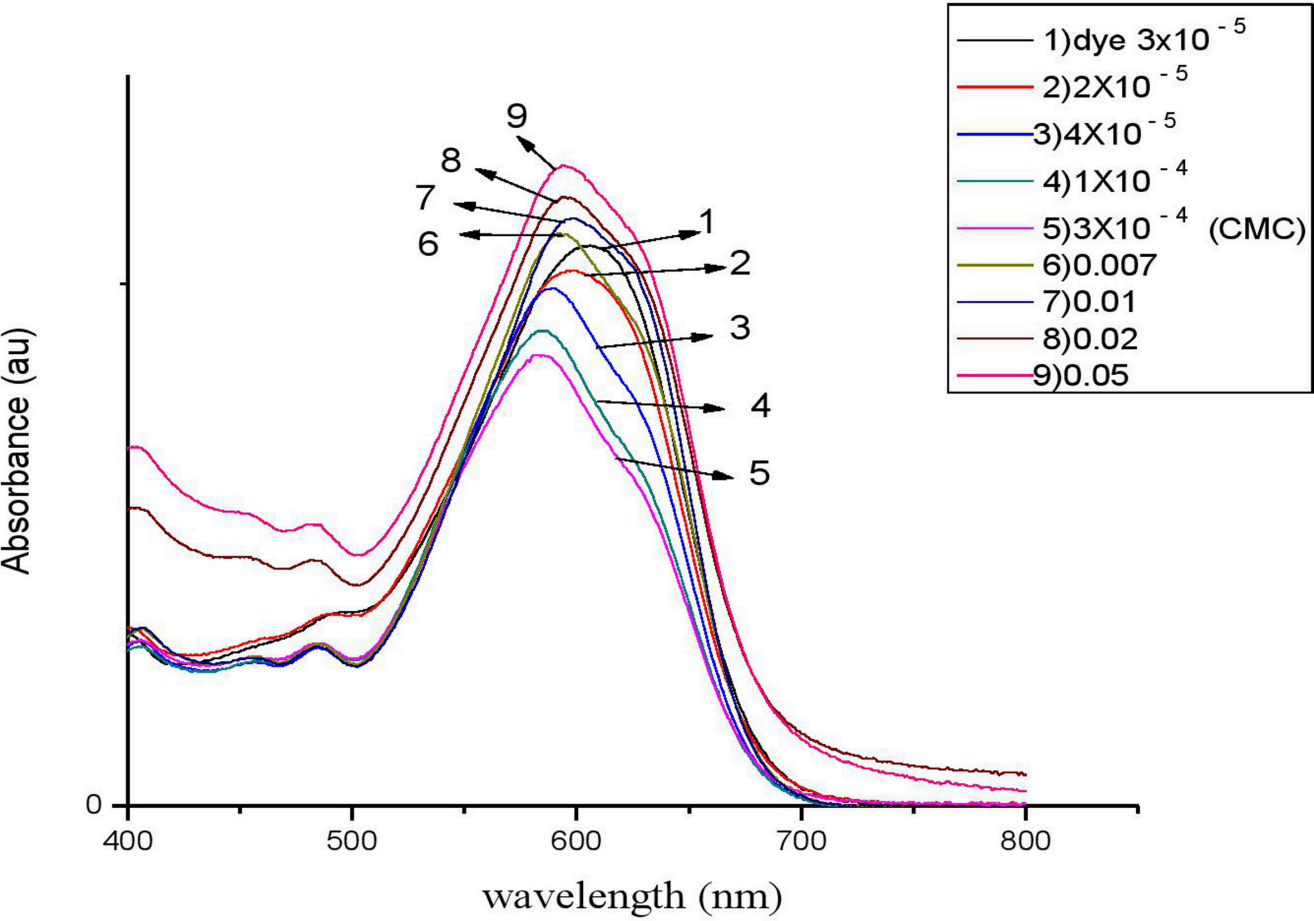
UV–visible absorption spectra of RBBR (3 × 10^−4^ M) associated with C_16_TAC at different concentrations.

**Figure 10.**
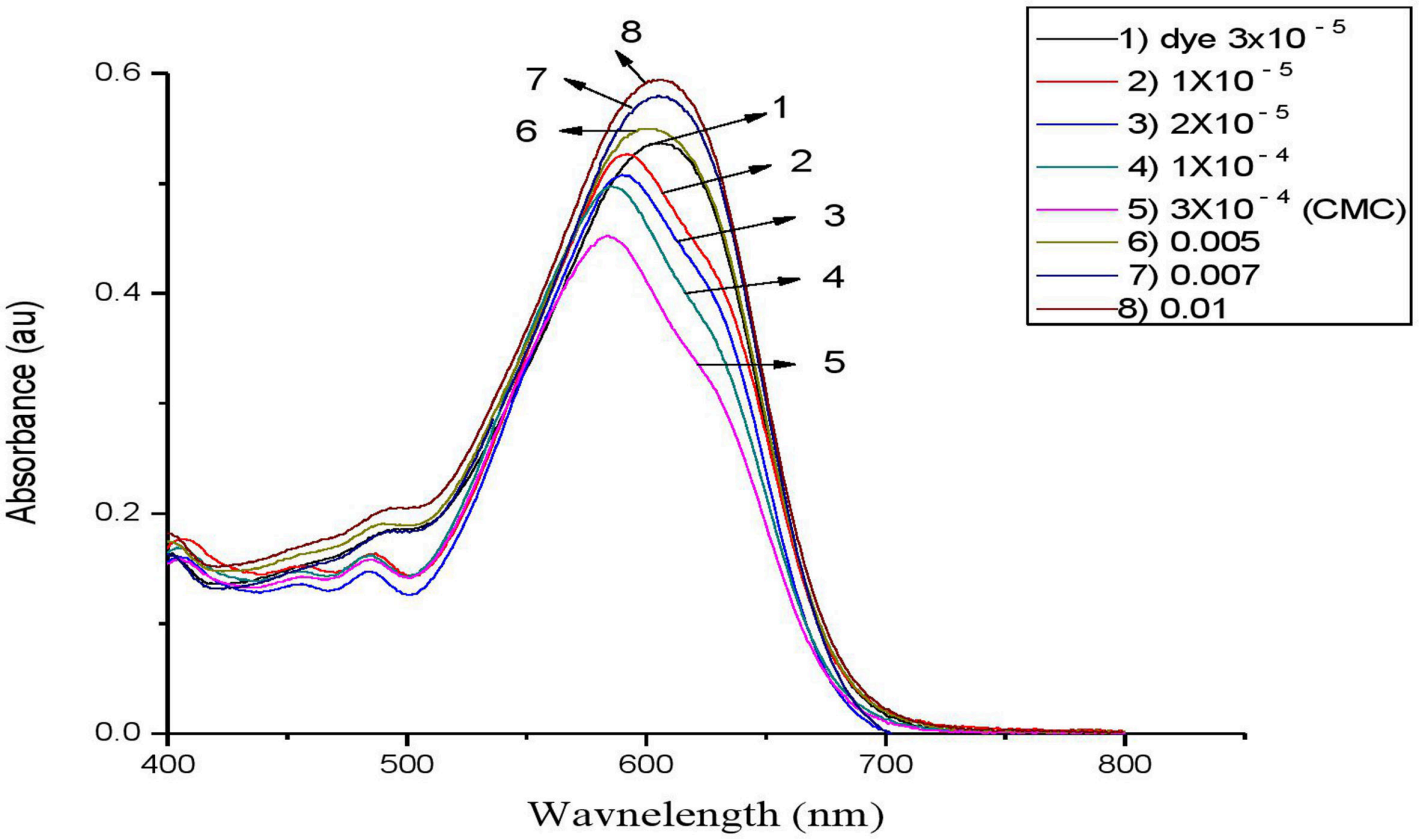
UV–visible absorption spectra of RBBR (3 × 10^−4^ M) associated with C_18_TAC at different concentrations.

Following our investigation, we examined the impact of C_*n*_TAC on the RBBR absorption spectra at room temperature. At 597 nm, the clean dye exhibits an absorption peak [67,68]. Figures 7–10 display the RBBR absorption spectra at 1.0 × 10^–5^ M when C_*n*_TAC concentrations range from 1.0 × 10^–5^ M to 0.1 M.

Interestingly, surfactants observed that the maximum absorption of RBBR at 575 nm decreased at concentrations lower than the CMC, and this decrease peaked at the CMC. The ion–ion interactions between RBBR and C_*n*_TAC lead to a decrease in absorption intensity. The presence of RBBR leads to a reduction in optical absorbance, resulting in a significantly lower CMC of C_*n*_TAC compared with that of the pure surfactant. This suggests that the interaction between C_*n*_TAC and RBBR affects the formation and stability of micelles, leading to a more efficient organization of surfactant molecules at lower concentrations. The observed decrease in CMC demonstrates the influence of the dye on the self-assembly behaviour of C_*n*_TAC and highlights the impact of the interaction on the overall system properties. A shift in maximum absorbance toward lower wavelengths is observed when dye molecules transition from aqueous to hydrophobic environments [69–73]. Such spectrum alterations in this particular case most likely result from interactions between C_*n*_TAC and RBBR inside micelles. With the concentration of C_*n*_TAC increasing from 0.02 to 0.1 M, above the CMC, there was a noticeable rise in absorbance. This surge indicates that dye molecules are integrated into C_*n*_TAC micelles, leading to the enlargement of the micelle core.

Interestingly, there is very little research on how changes in CMC affect the RBBR–C_*n*_TAC interaction. Because of the observable effect of alkyl groups (C_12_, C_14_, C_16_ and C_18_) on the decrease of absorbance value, C_*n*_TAC is essential in reducing CMC by reducing the absorbance. By facilitating a more compact arrangement of surfactant molecules within micelles, these hydrophobic moieties lower the CMC.

As observed in other dye–surfactant systems, the hydrophobic tails of C_*n*_TAC further stabilize micelles by interacting with RBBR’s hydrophobic domains; the shift in absorption wavelength indicates a dye transition into hydrophobic micelle cores; the ion–ion interactions between the surfactant’s positive head and the dye’s negative charge result in a reduction of CMC; the observed decrease in absorbance at lower C_*n*_TAC concentrations shows enhanced micelle formation and organization at reduced concentrations.

The main mode of interaction is probably driven by electrostatic forces between C_*n*_TAC head group (the positive charge) and RBBR (the negative charge).

Furthermore, it is possible that the hydrophobic tail of C_*n*_TAC interacts with the hydrophobic domains of RBBR in a way comparable to that of eosin-Y, carminic acid and Eriochrome Blue Black R [64,74,75]. Table 1 shows how the length of the carbon chain (hydrophobicity) in C_*n*_TAC surfactants influences two key aspects: their ability to increase dye solubility and the CMC.

**Table 1.** The CMC determined at approximately 101 325 Pa using equations (3.4) and (3.6) at 298.15 K and 101.3 kPa with uncertainty ±0.5.

	CMC (M) values obtained from spectroscopic measurements			
	C_12_TAC	C_14_TAC	C_16_TAC	C_18_TAC
absence of dye [45]	1.5 × 10^–2^	3.8 × 10^–3^	9.0 × 10^–4^	6.5 × 10^–4^
interaction with RBBR	7 × 10^–3^	1 × 10^–3^	3 × 10^–4^	3 × 10^–4^
interaction with carminic acid [54]	7.0 × 10^–3^	1.50 × 10^–3^	4 × 10^–4^	
interaction with eosin-Y [55]	3.0 × 10^–4^	1.0 × 10^–4^	8 × 10^–3^	

### 3.3. Benesi–Hildebrand equation for the determination of the binding constant between C*_n_*TAC and RBBR

The binding constant (*K*_b_) between the dye and surfactant was determined based on our spectrophotometric data. Our experiments allowed for the characterization of the interaction type and its strength. *K*_b_, the equilibrium constant, represents a specific value that indicates the stability of this reaction at equilibrium as equation (3.3):


(3.3) D+M→DM.

Here, D, M and DM stand for dye, micelle and dye–micelle complex, respectively. *K*_b_ is defined by the Benesi–Hildebrand equation (3.4) [76,77]:


(3.4) $$\frac{D_{T}}{\Delta A}=\frac{1}{\left[\varepsilon_{m}-\varepsilon_{0}\right]}+\frac{1}{K_{b}\left[\varepsilon_{m}-\varepsilon_{0}\right]C_{m}},$$

$where\;\varepsilon_{m}$ is the molar extinction coefficient of the dye–micelle complex (l mol^−1^ cm^−1^), *D*_T_ represents the concentration of RBBR (M) and ∆*A* = *A* – *A*_0_ denotes the change in RBBR absorbance observed after the addition of the surfactant. The concentration of the surfactant in its micellized form is represented as *C*_m_ (M), which can be calculated as the difference between the surfactant concentration (*C*_s_, M) and the CMC (M). In this equation, *ε*_0_ represents the molar extinction coefficient of RBBR (l mol^−1^ cm^−1^) and *K*_b_ represents the stability constant (l mol^−1^).

As an example, electronic supplementary material, figures S5–S8, show the plots of *D*_T_/∆*A* against 1/*C*_m_ for *C*_*n*_TAC. The slope and intercept of these linear plots are used to calculate the values of *K*_b_ and *ε*_m_, respectively.

According to the calculated *K*_b_ values in tables 2,3 and 3, the surfactant reactivity for RBBR increases as follows:

**Table 2.** The Benesi–Hildebrand equation utilized to determine the values of CMC, *K*_b_ and ∆*G* for the interaction between *C*_*n*_TAC and RBBR at 298.15 K and 101.3 kPa with uncertainty ±0.5.

CMC (M)	7.0 × 10^–3^	1.0 × 10^–3^	3.0 × 10^–4^	3.0 × 10^–4^
*K*_b_ (l mol^−1^)	48.31	74.07	171.36	452.18
∆*G* (kJ mol^−1^)	–9.607	–10.66	–12.74	–15.14

**Table 3.** The Scott equation utilized to determine the values of CMC, *K*_b_ and ∆*G* for the interaction between C_*n*_TAC and RBBR at 298.15 K and 101.3 kPa with uncertainty ±0.5.

CMC (M)	7.0 × 10^–3^	1.0 × 10^–3^	3.0 × 10^–4^	3.0 × 10^–4^
*K*_b_ (l mol^−1^)	23	43.71	87.08	388.69
∆*G* (kJ mol^−1^)	−7.77	−9.35	−11.06	−14.77


$$C_{18}TAC>C_{16}TAC>C_{14}TAC>C_{12}TAC.$$

The length of each hydrocarbon chain has a substantial impact on the mutual affinity and form of contact between the micelles of C_*n*_TAC and RBBR, as well as on the kinetics of their interaction.

Tables 2 and 3 show how variations in *K*_b_ are correlated, both directly and indirectly, with band shift and CMC. There is a clear correlation between hydrophobicity and *K*_b_. Furthermore, there is a clear association between the hydrophobicity of the surfactant and the CMC that results from the addition of RBBR.

The findings show that the hydrocarbon chain length of C_*n*_TAC has a major impact on the micelle affinity and on the kinetics of its interaction with RBBR. The binding constant (*K*_b_) is directly related to the surfactant’s hydrophobicity since micelle affinity for RBBR is enhanced by greater hydrophobicity. Improvements in micelle stability and organization are further demonstrated by band shifts and lower CMC values. Tables 2 and 3 demonstrate how the length of the surfactant chain affects these characteristics, with longer chains encouraging stronger interactions and lower CMCs. In order to improve dye solubility and self-assembly efficiency at lower surfactant concentrations, RBBR facilitates tighter micelle packing through hydrophobic and electrostatic interactions.

### 3.4. Determination of the binding constant by applying the Scott equation

To determine the binding constant and molar extinction coefficient, an alternative method is to utilize the modified Scott equation [78]. This approach provides an alternative approach for calculating these parameters as in equation (3.5):


(3.5) $$\frac{[D][S]l}{d-d_{0}}=\frac{\left[C\right]}{\varepsilon_{c}-\varepsilon_{0}}+\frac{1}{K_{b}[\varepsilon_{c}-\varepsilon_{0}]},$$

where [*D*] is the concentration of the dye (M), [*S*] is the concentration of the surfactant (M), [*C*] is the total concentration of the dye–surfactant complex (M) and *l* is the path length of the cell (cm).

When comparing the absorbances of the dye at the absorption maximum with and without the presence of the surfactant, *d* represents the molar extinction coefficient of the dye (l mol^−1^ cm^−1^), while *d*_0_ corresponds to the molar extinction coefficient of the complex formed between the dye and the surfactant (l mol^−1^ cm^−1^). Similarly, *ε*_c_ represents the molar extinction coefficient of the complex (l mol^−1^ cm^−1^) and *ε*_0_ represents the molar extinction coefficient of the dye alone (l mol^−1^ cm^−1^). Together, these characteristics help to provide a thorough understanding and quantitative analysis of the surfactant and dye interactions within the experimental context.

Empirical results are shown as [*D*][*C*]/Δ*A* against concentration in electronic supplementary material, figures S9–S12. The linearity of the plots makes it easier to determine *ε*_c_ and *K*_b_ from the slope and the intercept, respectively. The surfactant affinities are shown by analysis of the acquired *K*_b_ values (tables 2 and 3). C_18_TAC has the highest affinity, followed by C_16_TAC, C_14_TAC and C_12_TAC, respectively. This arrangement perfectly fits the hierarchical sequence of the Benesi–Hildebrand equation.

### 3.5. Gibbs free energy of micellization through Scott equation

To evaluate ∆*G* of RBBR’s micelle binding tendency, the following equation was applied (equation (3.6)):


(3.6) $$\Delta G=-RT\ln K_{b}.$$

The study emphasizes the significance of representing the universal gas constant and the absolute temperature as important factors in the investigation. Nazar and co-workers have done a study between dyes and surfactants and their notable contributions have been published in the literature [79–81]. Significant interactions between the dye and surfactant molecules are shown by the observed decrease in dye absorption upon surfactant addition. The reduced binding constant of C_*n*_TAC may be explained by hydrophobic interactions, as it has a propensity to form inverse micelles in which its non-polar segments extend into the aqueous phase.

Tables 2 and 3 clearly show a trend where the binding constant rises as the alkyl chain lengthens, supporting the idea that molecular structure affects the interactions that are seen. These results provide important new information about the fundamental principles that govern regulating dye–surfactant interactions, which advances our understanding of these intricate systems in the field of chemical research.

The importance of the absolute temperature and universal gas constant in comprehending dye–surfactant interactions is emphasized by this work. The observed decrease in dye absorption upon the addition of C_*n*_TAC suggests important interactions, mostly due to hydrophobic forces. The propensity of C_*n*_TAC to form inverse micelles, in which non-polar segments protrude into the aqueous phase, is thought to be the cause of its decreased binding constant. Alkyl chain length and binding constant are directly correlated, as seen in tables 2 and 3, illustrating the role that molecular structure plays in these relationships. Hydrophobicity is improved by longer alkyl chains, which also increase the binding constant and fortify micelle–dye interactions. These results reveal important new information on the fundamentals of dye–surfactant systems, with wider ramifications for comprehending and improving chemical interactions in intricate systems.

### 3.6. Estimating the Gibbs free energy from the binding constant using the Benesi–Hildebrand equation

The Benesi–Hildebrand equation showed that the ∆*G* observed in RBBR–C_*n*_TAC systems and C_*n*_TAC systems was similar. Tables 2 and 3 display the calculated values of *K*_b_ (binding constant) and standard ∆*G* obtained from the two calculations. The results indicate that the C_*n*_TAC systems exhibit smoother and more successful interactions [82]. The results show that the longer the carbon chains in the surfactant molecules, the more negative ∆*G* becomes. This indicates that binding between the dye and surfactant becomes more favourable (spontaneous) with longer chains, which aligns with what other research has found. The attraction between the dye RBBR and the surfactant C_*n*_TAC (*N*-cetyl-*N*,*N*,*N*-trimethylammonium chloride) is affected by how well they distribute electrical charge. In the case of compounds like RBBR, C_*n*_TAC acts as a host, capable of entrapping dye molecules through polar interactions with specific ions like N^+^(CH_₃_)_₃_Cl^−^ or SO_3_^2−^. At the same time, the water-hating tail of C_*n*_TAC interacts with the dye’s water-hating parts. This interaction can cause the dye to absorb less light, leading to lower absorbance levels [83].

Similar ∆*G* values are found for RBBR–C_*n*_TAC and pure C_*n*_TAC systems using the Benesi–Hildebrand equation, suggesting efficient and smooth interactions. As can be seen in tables 2 and 3, longer alkyl chains provide greater negative ∆*G* values, which indicate more advantageous (spontaneous) dye–surfactant binding. With C_*n*_TAC functioning as a host and ensnaring dye molecules through polar and hydrophobic interactions, the electrical charge distribution in RBBR and C_*n*_TAC affects how they interact. Lower dye absorption levels are correlated with these interactions, which decrease light absorbance. Even in situations devoid of SO_3_^2−^ or –OH groups, empirical evidence indicates that hydrogen bonding and repulsive interactions have an additional impact on C_*n*_TAC surfactants’ ability to modify dye behaviour. Our knowledge of these intricate systems is improved by these discoveries, which offer crucial insights into the mechanics behind dye–surfactant interactions.

Furthermore, empirical data indicate that the cationic surfactant C_*n*_TAC, which plays a crucial role in the promotion of hydrogen bonding, can induce colour shifts in certain indicators even when the functional groups SO_3_^2−^ and –OH are absent in alcohol or water environments. This phenomenon is likely attributed to hydrogen bonding or repulsive negative interactions occurring. This interpretation sheds light on the intricate relationships between C_*n*_TAC and its subsequent impact on dye behaviour. It presents valuable new insights into the underlying mechanisms that govern such events and offers important information on the fundamental understanding of these phenomena.

## 4. Conclusions

The study examined the micelle generation of C_*n*_TAC surfactants in water with different alkyl chain lengths (*n* = 12, 14, 16 and 18). In line with earlier research, conductometric measurements showed that the CMC falls as alkyl chain length increases. Electrostatic interactions between the quaternary ammonium group (–N^+^(CH_3_)_3_Cl^−^) of C_*n*_TAC and the sulfonate group (SO_3_^2−^) of RBBR resulted in dye encapsulation in micelles, as evidenced by the noticeable blue shift in absorption variations of the anionic dye RBBR in the presence of C_*n*_TAC.

As alkyl chain length grew, the binding constant—which was determined using the Benesi–Hildebrand equation—decreased, indicating weaker interactions between RBBR and micelles made of longer-chain C_*n*_TAC. Reduced absorbance was the result of C_*n*_TAC surfactants’ disruption of RBBR molecular complexes at lower doses. Stable dye–surfactant complexes were created at greater concentrations, improving the efficiency of light absorption. These changes are the result of a dynamic balance between the electrostatic forces between the dye and surfactant and the hydrophobic contacts of the alkyl chain. The spontaneous nature of these interactions was validated by negative Gibbs free energy values (Δ*G*). The influence of hydrophobic chain length on micellization and dye contact was highlighted by the fact that longer alkyl chains produced larger negative Δ*G* values.

## Data Availability

Data that support this study have been uploaded as electronic supplementary material [84].
